# Plasma Transthyretin Levels and Risk of Type 2 Diabetes Mellitus and Impaired Glucose Regulation in a Chinese Population

**DOI:** 10.3390/nu14142953

**Published:** 2022-07-19

**Authors:** Xiaoli Hu, Qianqian Guo, Xiaoqian Wang, Qiang Wang, Liangkai Chen, Taoping Sun, Peiyun Li, Zhilei Shan, Liegang Liu, Chao Gao, Ying Rong

**Affiliations:** 1 Department of Nutrition, Wuchang Hospital Affiliated to Wuhan University of Science and Technology, 116 Yangyuan Street, Wuhan 430063, China; hxlfox1993@163.com; 2 Department of Nutrition and Food Hygiene, Hubei Key Laboratory of Food Nutrition and Safety, School of Public Health, Tongji Medical College, Huazhong University of Science and Technology, 13 Hangkong Road, Wuhan 430030, China; m201775163@hust.edu.cn (Q.G.); wxq2001023@163.com (X.W.); d201981402@hust.edu.cn (Q.W.); clk@hust.edu.cn (L.C.); D201578100@alumni.hust.edu.cn (T.S.); lipeiyunhuster@126.com (P.L.); zhileishan@hust.edu.cn (Z.S.); lgliu@mails.tjmu.edu.cn (L.L.); 3 MOE Key Laboratory of Environment and Health, School of Public Health, Tongji Medical College, Huazhong University of Science and Technology, 13 Hangkong Road, Wuhan 430030, China; 4 National Institute for Nutrition and Health, Chinese Center for Disease Control and Prevention, 27 Nanwei Road, Beijing 100050, China; gaochao20090901@163.com; 5 Department of Clinical Nutrition, Shandong Provincial Hospital, Shandong University, 324 Five Weft Seven Road, Jinan 250021, China; 6 Department of Clinical Nutrition, Shandong Provincial Hospital Affiliated to Shandong First Medical University, 324 Five Weft Seven Road, Jinan 250021, China

**Keywords:** type 2 diabetes mellitus, impaired glucose regulation, transthyretin, case-control study

## Abstract

Plasma transthyretin may be engaged in glucose regulation. We aimed to investigate the association between plasma transthyretin levels and the risk of newly diagnosed T2DM and impaired glucose regulation (IGR) in a Chinese population. We conducted a case-control study including 1244 newly diagnosed T2DM patients, 837 newly diagnosed IGR patients, and 1244 individuals with normal glucose tolerance (NGT) matched by sex and age. Multivariate logistic regression analysis was utilized to estimate the independent association of plasma transthyretin concentrations with the risk of T2DM and IGR. Plasma transthyretin concentrations were significantly higher in T2DM and IGR patients compared with control subjects (*p* < 0.005). After multiple adjustment and comparison with the lowest quartile of plasma transthyretin concentrations, the odds ratios (95% confidence intervals) of T2DM and IGR in the highest quartile were 2.22 (1.66, 2.98) and 2.29 (1.72, 3.05), respectively. Plasma transthyretin concentrations also showed a great performance in predicting the risk of T2DM (AUC: 0.76). Moreover, a potential nonlinear trend was observed. Our results demonstrated that higher plasma transthyretin concentrations, especially more than 290 mg/L, were associated with an increased risk of T2DM and IGR. Further studies are warranted to confirm our findings and elucidate the potential mechanisms.

## 1. Introduction

Type 2 diabetes mellitus (T2DM), characterized by dysregulation of glucose homeostasis [1,2], is a growing concern worldwide. The prevalence of T2DM has dramatically increased over the last few decades, resulting in a large social and economic burden. Even worse, according to the latest data from International Diabetes Federation, 537 million adults (20–79 years) are living with diabetes. This number is predicted to rise to 643 million by 2030 and 783 million by 2045 [3].

Transthyretin, formerly referred to as prealbumin, is a 55kDa tetrameric protein composed of four identical subunits and acts as a direct transport protein for thyroxine and indirect transport protein for retinol [4,5,6]. It is mainly synthesized by liver and choroid plexus epithelial cells and exists in plasma and/or cerebrospinal fluid of vertebrates [7,8]. Conventionally, a higher serum transthyretin level is usually perceived as a sensitive clinical index of better protein nutritional status [9,10].

Recently, transthyretin was found to be synthesized within pancreatic islets, stored in the secretory vesicle, and plays an important role in the secretion of insulin and glucagon [11,12,13,14]. Moreover, animal studies have shown that lowering transthyretin levels could obviously improve insulin sensitivity in obesity and T2DM [15,16]. These findings suggested that transthyretin may be engaged in the initiation and development of T2DM. However, studies exploring transthyretin levels under T2DM condition did not reach an agreement [17,18,19,20,21,22,23]. Small sample size and not well-matched enrollment may be responsible for the above discrepancy.

Therefore, the objective of our study was to investigate the association between plasma transthyretin levels and the risk of T2DM and impaired glucose regulation (IGR) in a relatively large-scale case-control study conducted among the hospital-based general population.

## 2. Materials and Methods

### 2.1. Study Population

The study population consisted of 3325 participants, including 1244 newly diagnosed T2DM patients, 837 newly diagnosed IGR patients, and 1244 individuals with normal glucose tolerance (NGT). The study population of recruitment had been described in detail in our previous publications [24,25]. Briefly, all cases were consecutively recruited from patients who, for the first time, received a diagnosis of T2DM in the Department of Endocrinology, Tongji Hospital, Tongji Medical College, Wuhan, China, from December 2010 to December 2016. Concomitantly, the general population undergoing a routine health checkup in the same hospital were enrolled as controls. The inclusion criteria were age ≥ 30 years, BMI < 40 kg/m^2^, and no history of diagnosis of diabetes or receiving pharmacological treatment for hypertension and hyperlipidemia. Participants with clinically significant neurological, endocrinological, psychological, or other systemic diseases, as well as acute illness or chronic inflammatory or infectious diseases, were excluded from the present study. A total of 837 newly diagnosed IGR patients also met the inclusion and exclusion criteria. In addition, cases were stringently matched to controls in a 1:1 ratio, based on sex and age (±3 years). All participants enrolled were of Han ethnicity and gave a commitment to take no medication known to affect glucose tolerance or insulin secretion before participating in the study. Written informed consent was obtained from each participant. The study was approved by the ethics committee of Shandong Provincial Hospital (NSFC:NO.2019-051).

### 2.2. Definition of T2DM and IGR

The diagnostic criteria were recommended by the World Health Organization in 1999, incorporating both fasting plasma glucose (FPG) and oral glucose tolerance test (OGTT) [26]. T2DM was confirmed when FPG ≥ 7.0 mmol/L and/or 2 h post-glucose load (OGTT2h) ≥ 11.1 mmol/L. IGR was defined as impaired fasting glucose ([FPG] ≥ 6.1 mmol/L and < 7.0 mmol/L, and [OGTT2h] < 7.8 mmol/L) and/or impaired glucose tolerance (FPG < 7.0 mmol/L, and OGTT2h ≥ 7.8 mmol/L and < 11.1 mmol/L). Those with FPG < 6.1 mmol/L and OGTT2h < 7.8 mmol/L were considered NGT.

### 2.3. Body Composition and Blood Parameters

Demographic information, including sex, age, family history of diabetes, smoking status, drinking status, history of hypertension, history of hyperlipidemia, and physical activity, was collected via face-to-face questionnaires. Anthropometric measurements, including waist circumference (cm), height (m), and weight (kg), were obtained by trained staff using standardized techniques. Body mass index (BMI) was calculated as weight divided by the square of height (kg/m^2^). After a 10 h overnight fast, all participants underwent a 75 g OGTT, with venous blood samples collected at 0 and 2 h for determination of FPG, fasting plasma insulin (FPI), and OGTT 2 h. Fasting blood samples were collected in EDTA-anticoagulative tubes and separated for plasma within 1 h. Plasma was then kept at −80 °C; prior to analysis. Plasma biochemical indices, such as total cholesterol (TC), triglyceride (TG), high-density lipoprotein cholesterol (HDL-C), and low-density lipoprotein cholesterol (LDL-C), were determined as described in our previous study [27]. Homoeostasis model assessment of insulin resistance (HOMA-IR) was evaluated using the formula: FPI (μU/mL) * FPG (mmol/L)/22.5. Homoeostasis model assessment of β-cell function (HOMA-β) was calculated as [20 * FPI (μU/mL)]/[FPG (mmol/L) − 3.5].

Plasma total protein, albumin, and transthyretin levels were measured by BS 200 Autoanalyser (Mindray, Shenzhen, China) using the biuret method, bromcresol green dye-binding method, and immunoturbidimetric method, respectively. In order to guarantee the stability and reliability of our data, reference standards were applied per 36 samples. The intra-day and inter-day coefficients of variation were both <3.5%.

### 2.4. Statistical Analysis

General demographic and laboratory characteristics were presented as mean ± standard deviation or median (interquartile) for continuous variables, and as frequency or percentage for categorical variables. A chi-square test (categorical variables), Student’s t-test (continuous variables, normal distribution), or Mann–Whitney U test (continuous variables, skewed distribution) were used to assess the differences of basic characteristics and biochemical indices between groups. As continuous variables, plasma transthyretin concentrations were categorized into quartiles according to the distribution of NGT group to calculate the odds ratios (ORs) of T2DM and IGR: Q1, < 189.71 mg/L; Q2, 189.71–224.44 mg/L; Q3, 224.44–264.11 mg/L; Q4, ≥ 264.11 mg/L, respectively. Multiple logistic regression analysis was performed to examine the independent association of plasma transthyretin with the risk of T2DM and IGR. Conventional risk factors of T2DM, consisting of sex, age, BMI, waist circumference, family history of diabetes, history of hypertension, history of hyperlipidemia, smoking status (yes or no), drinking status (yes or no), and physical activity (at least once/week or no), were adjusted. To minimize the impact of nutritional status, we further adjusted plasma total protein and albumin levels. The linear trend across increasing transthyretin quartiles was tested by assigning the median value to each quartile and treating it as a continuous variable. To estimate the coherence of the findings in different subgroups, we performed stratified analyses by sex, age (<60 years and ≥60 years), BMI (<24 kg/m^2^ and ≥24 kg/m^2^), family history of diabetes, smoking status, drinking status, history of hypertension, history of hyperlipidemia, and physical activity. The predictive power of the model including plasma transthyretin concentrations was tested using receiver operating characteristic (ROC) curve analysis. We further conducted a restricted cubic spline with four knots at the 20th, 40th, 60th, and 80th percentiles of plasma transthyretin concentrations to evaluate a potential nonlinear relationship between plasma transthyretin and the risk of T2DM and IGR, excluding values outside the 1st and 99th percentiles. Assuming the control group proportion of transthyretin exposure was 0.25 and the OR was 1.47 [21], comparing the highest with the lowest quartile of transthyretin, our study had more than 90% power to detect the difference.

All analyses were performed using the Windows-based SPSS 24.0 (SPSS Inc., Chicago, IL, USA) and Stata/MP 14.0 (StataCorp LP, College Station, TX, USA). All *p* values presented were two-tailed with a significance level of 0.05.

## 3. Results

General demographic and clinical characteristics of the 3325 participants are summarized in Table 1. In comparison with controls, T2DM and IGR individuals had higher BMI and waist circumference, greater prevalence of family history of diabetes, history of hypertension and hyperlipidemia, and higher levels of TC, TG, LDL-C, FPG, OGTT2h, and FPI, but lower levels of HDL-C. Plasma transthyretin concentrations were significantly higher in T2DM and IGR patients compared with the control subjects (229.01 ± 53.69 mg/L, 246.97 ± 64.77 mg/L, and 246.50 ± 63.01 mg/L in the NGT, T2DM, and IGR groups, respectively, *p* < 0.005).

Data are presented as a number (percentage) for categorical data, mean (standard deviation) for parametrically distributed data, or median (interquartile range) for nonparametrically distributed data. Abbreviations: BMI, body mass index; FPG, fasting plasma glucose; FPI, fasting plasma insulin; HDL-C, high-density lipoprotein cholesterol; HOMA-β, homeostasis model assessment of β-cell function; HOMA-IR, homeostasis model assessment of insulin resistance; IGR, impaired glucose regulation; LDL-C, low-density lipoprotein cholesterol; NGT, normal glucose tolerance; T2DM, type 2 diabetes mellitus; TC, total cholesterol; TG, triglyceride. Table 2 presents logistic regression results for T2DM and IGR associated with plasma transthyretin concentrations, categorized into quartiles based on the distribution in controls. Comparing the highest with the lowest quartile of transthyretin, the crude ORs (95% confidence intervals, CIs) of T2DM and IGR were 2.23 (1.76, 2.82) and 2.00 (1.56, 2.56), respectively. Similar result was obtained in the T2DM and IGR combined group, with a crude OR (95% CI) of 2.02 (1.65, 2.46). Adjustment for sex, age, BMI, waist circumference, family history of diabetes, smoking status, drinking status, history of hypertension, history of hyperlipidemia, and physical activity did not change the observed association substantially. The above results were likewise robust after further adjustment of protein nutritional status, including plasma total protein and albumin levels. We then combined the IGR and NGT groups as a control group, with an OR (95% CI) of T2DM of 1.72 (1.37, 2.14) after a full adjustment. In stratified analyses, significant interactions were found between plasma transthyretin concentrations and sex (*p* = 0.022), BMI (*p* = 0.023), smoking status (*p* = 0.007), and drinking status (*p* = 0.024). Compared with men, women with higher transthyretin concentrations possessed an increased risk of T2DM. Similarly, individuals with a lower BMI (<24 kg/m^2^) and higher transthyretin levels had a higher risk of T2DM in comparison with those with a higher BMI (≥24 kg/m^2^). Moreover, significant relationships between plasma transthyretin concentrations and risk of T2DM were observed among nonsmokers and nondrinkers, whereas the above associations vanished with respect to smokers and drinkers (Table 3).

**Figure 1:**
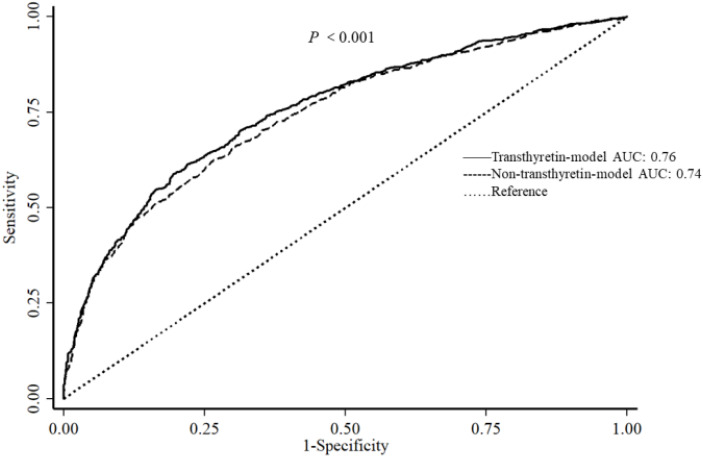
ROC curve of transthyretin-model and non-transthyretin-model. Abbreviations: AUC: area under the curve; ROC, receiver operating characteristic.

**Figure 2:**
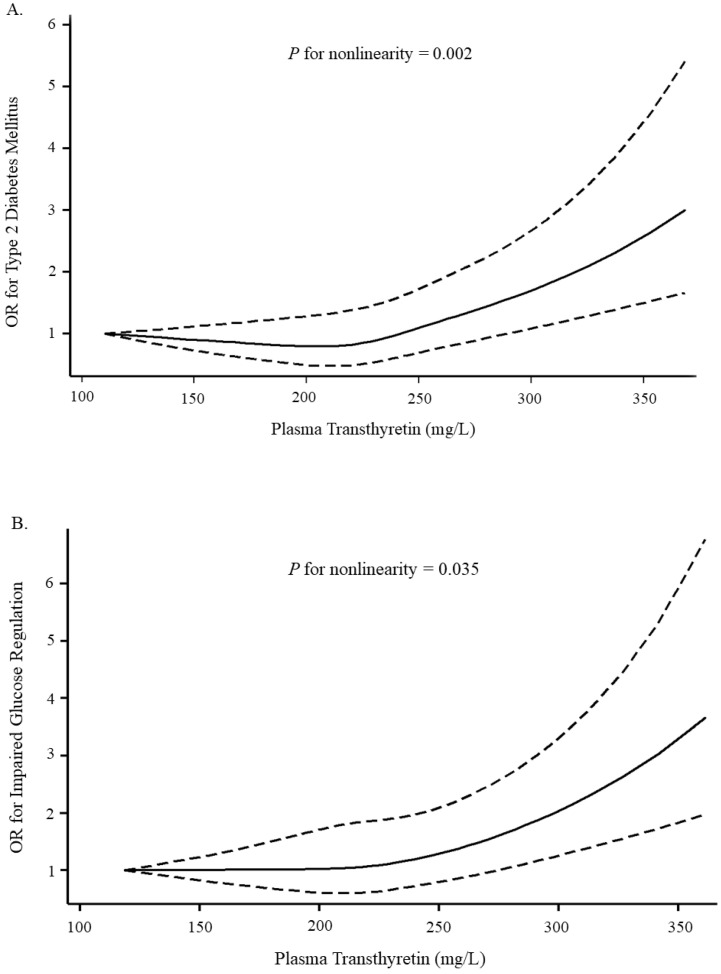
Representation of restricted cubic spline logistic regression models for plasma transthyretin concentrations and risk of T2DM (**A**) and IGR (**B**). Results were adjusted for sex, age, body mass index, waist circumference, family history of diabetes, smoking status, drinking status, history of hypertension, history of hyperlipidemia, physical activity, and total protein and albumin levels. (ORs, solid line; 95% Cis, dashed line). Abbreviations: IGR, impaired glucose regulation; OR, odds ratio; T2DM, type 2 diabetes mellitus.

The areas under the ROC curve for transthyretin-model and non-transthyretin-model were 0.76 and 0.74, respectively, confirming that the predictive power of transthyretin-model was better (*p* < 0.001), despite small sample sizes (Figure 1). The spline regression analysis indicated a potential nonlinear relationship between plasma transthyretin levels and T2DM (*p* for nonlinearity = 0.002) (Figure 2A). A similar curve was generated in the association between plasma transthyretin levels and IGR (*p* for nonlinearity = 0.035) (Figure 2B). Higher plasma transthyretin concentrations, especially more than 290 mg/L, were associated with an increased risk of T2DM and IGR.

## 4. Discussion

To the best of our knowledge, this was the first study to report the association between plasma transthyretin levels and the risk of newly diagnosed T2DM and IGR in a relatively large-scale Chinese population. We found that plasma transthyretin levels were positively associated with the risk of T2DM and IGR in a nonlinear dose–response manner. When plasma transthyretin levels reached 290 mg/L, which was still in the normal range, a significant increased risk of T2DM and IGR was observed. We considered that higher plasma transthyretin levels may not only represent a better nutritional status, but also imply a higher risk of T2DM and IGR. Considering the facilitation and accessibility, plasma transthyretin may be a promising biomarker for T2DM and IGR in the future.

Our findings were in accordance with some previous epidemiological studies. Chen and colleagues conducted a cross-sectional study, which recruited 10,309 participants aged 40 years or above from Shanghai, China, and demonstrated that elevated serum transthyretin levels were associated with increased risks of T2DM [21]. Similarly, small-scale case-control studies in Asian Indians and Caucasians supported the aforementioned result [17,22]. With a proteomic-based approach, researchers found that transthyretin levels were up-regulated in subjects with T2DM [23]. On the other hand, some studies indicated that circulating transthyretin did not differ between groups of controls and T2DM cases [18,19]. On the contrary, by using surface enhanced laser desorption/ionization time-of-flight mass spectrometry, transthyretin was found to be lower in serum of T2DM patients in comparison with normal individuals [20]. Apart from detection methods, differences in population included, definition of T2DM and outcomes, and inadequate adjustment of potential confounding factors may have contributed to the above discrepancies. Since these studies were not limited to newly developed cases, lifestyle changes and medication may interfere with the causal link between exposure and outcome. In addition, a detailed dose–response description was not available in the aforementioned studies.

With a large sample size, we conducted further subgroup analyses. Significant interactions were found between plasma transthyretin concentrations and sex, BMI, smoking status, and drinking status. Sex steroid hormones, such as 17β-estradiol (E2) and 5α-dihydrotestosterone (DHT), had been shown to take part in the regulation of transthyretin [28,29]. As a regulator of transthyretin mRNA expression, DHT had a greater role in transthyretin synthesis than E2 in female mice [30]. Under normal conditions, sex differences of transthyretin concentrations existed. At the onset of puberty, males had a more pronounced elevation of transthyretin than females. The influence of sex steroid hormones on transthyretin synthesis and/or turnover rate had been confirmed in several clinical conditions [5]. Therefore, differences of sex hormones may partly be responsible for the sex distinction in the relationship between plasma transthyretin levels and T2DM. Nevertheless, other interactions had not previously been reported and remain to be verified in future.

Our findings considered that higher plasma transthyretin concentrations, especially more than 290 mg/L, were positively associated with the risk of T2DM and IGR. Although the underlying mechanisms remain to be elucidated, some potential explanations may be biologically plausible. Firstly, transthyretin might play an important role in glucose homeostasis via regulating glucagon expression. Compared to wild-type mice, transthyretin-knockout mice showed significantly lower glucagon content, whether fasting or after insulin injection. In contrast, overexpression of transthyretin by using plasmid significantly increased glucagon mRNA expression in PANC-1 cells [13]. Secondly, transthyretin may disturb glucose homeostasis by inducing the aggregation of islet amyloid polypeptide (IAPP), which in turn led to the damage of pancreatic β-cells [11]. With the use of immunohistochemistry and in situ hybridization, Westermark and colleagues found that a higher transthyretin immunoreactivity of β-cells was associated with heavier amyloid deposits in pancreatic islets. Meanwhile, IAPP fibrils were considered to be interacted with transthyretin, which further resulted in amyloid fibrosis deposition and eventually gave rise to the decreased percentage of pancreatic β-cells. Thirdly, transthyretin tetramer constitutes a component in normal β-cell function, through promoting glucose-induced increases in cytoplasmic free Ca2+ concentration and insulin release, accompanied by protecting against β-cell apoptosis. However, transthyretin monomer was without the above effect. Unfortunately, Refai and colleagues discovered that serum transthyretin tetramer concentration was decreased, whereas the monomeric form was increased in patients with type 1 diabetes mellitus [12,14]. Whether the aforementioned situation is applicable to T2DM remains to be explored. Last but not least, transthyretin may cause diabetes by altering retinol binding protein 4 (RBP4)-transthyretin binding. Circulating in the blood, RBP4 complexed with retinol and bound to transthyretin with a high affinity [31]. Increased transthyretin or alterations in RBP4-transthyretin binding may lead to insulin resistance by stabilizing RBP4 at a higher steady-state concentration in circulation [16]. The unfavorable effects of transthyretin on T2DM could be partly attributed to indirectly elevated contents of RBP4, which appeared to be positively associated with prediabetes, T2DM, and other obesity-associated diseases in various animal and epidemiologic studies [32,33,34,35,36]. Overall, the specific mechanism remains to be further explored.

Large sample size was a prominent strength of our study by significantly increasing the statistical power to detect potential associations. In addition, our cases were confined to the newly diagnosed and drug-naïve patients, in order to avert possible changes in diet and lifestyle as well as medication, which may interfere with the results. Furthermore, multivariate adjusted models were utilized to reduce the impact of potential confounding factors.

Nonetheless, several limitations of our study should also be acknowledged. Firstly, the case-control nature of our study did not allow us to deduce any causality between plasma transthyretin and T2DM. Further studies are urgently needed to explore the above relationship. Secondly, despite having carefully adjusted for substantial potential confounders, other related factors that might affect our results cannot be ruled out. We also lacked information on dietary exposure, inflammation markers, and plasma RBP4 plus glucagon levels, which might also bias our results. Finally, our participants were of Chinese Han ethnicity and the results may not be easily extrapolated to other populations.

## 5. Conclusions

Our study demonstrated that higher plasma transthyretin concentrations, especially more than 290 mg/L, were associated with an increased risk of T2DM and IGR in a Chinese population. Further studies are warranted to confirm our results and explore the underlying mechanisms.

## Figures and Tables

**Table 1:** Demographic and clinical characteristics of NGT, T2DM, and IGR groups.

Parameters	NGT *(n* = 1244)	T2DM (*n* = 1244)	IGR (*n* = 837)	*p* Value	
				T2DM vs. NGT	IGR vs. NGT
Age (y)	50.88 (10.59)	51.08 (10.59)	51.45 (11.15)	0.640	0.237
Male, *N* (%)	728 (58.52%)	728 (58.52%)	503 (60.10%)	1	0.474
BMI (kg/m^2^)	23.59 (3.02)	25.30 (3.46)	24.94 (3.36)	<0.001	<0.001
Waist circumference (cm)	83.01 (9.14)	86.96 (10.69)	85.79 (9.95)	<0.001	<0.001
FPG (mmol/L)	5.57 (5.28–5.83)	8.08 (7.15–10.70)	6.29 (6.10–6.58)	<0.001	<0.001
FPI (μU/mL)	7.43 (5.08–11.08)	10.17 (6.87–15.23)	9.62 (6.52–13.94)	<0.001	<0.001
OGTT2h (mmol/L)	6.59 (5.79–7.33)	16.03 (12.81–20.37)	8.61 (7.49–9.66)	<0.001	<0.001
HOMA-IR	1.82 (1.25–2.72)	4.10 (2.61–6.15)	2.69 (1.81–3.87)	<0.001	<0.001
HOMA-β	74.19 (51.00–109.61)	43.75 (24.38–70.34)	68.91 (47.98–103.86)	<0.001	0.017
TG (mmol/L)	1.41 (1.06–1.77)	1.60 (0.96–2.72)	1.46 (0.95–2.34)	<0.001	0.037
TC (mmol/L)	4.36 (3.84–4.91)	4.87 (3.86–5.91)	4.68 (3.84–5.46)	<0.001	<0.001
HDL-C (mmol/L)	1.35 (1.21–1.51)	1.14 (0.87–1.49)	1.28 (0.99–1.56)	<0.001	<0.001
LDL-C (mmol/L)	2.18 (1.62–2.93)	2.87 (1.97–3.83)	2.72 (1.84–3.44)	<0.001	<0.001
History of hypertension, *N* (%)	237 (19.05%)	435 (34.97%)	266 (31.78%)	<0.001	<0.001
History of hyperlipidemia, *N* (%)	244 (19.61%)	511 (41.08%)	280 (33.45%)	<0.001	<0.001
Current smoker, *N* (%)	406 (33.49%)	322 (25.88%)	213 (25.45%)	<0.001	<0.001
Current drinker, *N* (%)	348 (27.97%)	323 (25.96%)	236 (28.20%)	0.259	0.912
Family history of diabetes, *N* (%)	116 (9.32%)	327 (26.29%)	154 (18.40%)	<0.001	<0.001
Physical activity (at least once/week), *N* (%)	516 (41.48%)	443 (35.61%)	321 (38.35%)	0.003	0.154
Plasma total protein (g/L)	74.05 (7.97)	73.70 (7.05)	74.13 (8.64)	0.272	0.816
Plasma albumin (g/L)	47.74 (5.80)	46.52 (5.09)	47.18 (6.04)	<0.001	0.100
Plasma transthyretin (mg/L)	229.01 (53.69)	246.97 (64.77)	246.50 (63.01)	<0.001	<0.001

**Table 2:** Odds ratios of T2DM and IGR, by quartiles of plasma transthyretin concentrations.

Groups	Quartile of Plasma Transthyretin Concentrations (mg/L)				*p* for Trend
	Q1 (Referent)	Q2	Q3	Q4	
	<189.71	189.71–224.44	224.44–264.11	≥264.11	
T2DM vs. NGT					
Cases/control subjects, *n*	238/311	243/311	281/311	482/311	
Crude OR (95% CI)	1	1.03 (0.81, 1.31)	1.24 (0.98, 1.57)	2.23 (1.76, 2.82)	<0.001
Adjusted OR^1^ (95% CI)	1	1.02 (0.77, 1.33)	1.23 (0.94, 1.61)	2.05 (1.58, 2.68)	<0.001
Adjusted OR^2^ (95% CI)	1	0.95 (0.72, 1.26)	1.10 (0.83, 1.46)	1.89 (1.43, 2.50)	<0.001
Adjusted OR^3^ (95% CI)	1	1.02 (0.76, 1.36)	1.24 (0.93, 1.66)	2.22 (1.66, 2.98)	<0.001
IGR vs. NGT					
Cases/control subjects, *n*	160/311	182/311	175/311	320/311	
Crude OR (95% CI)	1	1.14 (0.87, 1.48)	1.09 (0.84, 1.43)	2.00 (1.56, 2.56)	<0.001
Adjusted OR^1^ (95% CI)	1	1.14 (0.87, 1.50)	1.13 (0.85, 1.49)	2.16 (1.65, 2.84)	<0.001
Adjusted OR^2^ (95% CI)	1	1.14 (0.87, 1.51)	1.09 (0.82, 1.45)	2.11 (1.60, 2.79)	<0.001
Adjusted OR^3^ (95% CI)	1	1.19 (0.90, 1.57)	1.15 (0.86, 1.54)	2.29 (1.72, 3.05)	<0.001
(T2DM&IGR) vs. NGT					
Cases/control subjects, *n*	398/311	425/311	456/311	802/311	
Crude OR (95% CI)	1	1.07 (0.87, 1.32)	1.15 (0.93, 1.41)	2.02 (1.65, 2.46)	<0.001
Adjusted OR^1^ (95% CI)	1	1.08 (0.87, 1.34)	1.19 (0.95, 1.48)	2.12 (1.71, 2.64)	<0.001
Adjusted OR^2^ (95% CI)	1	1.06 (0.85, 1.33)	1.13 (0.90, 1.41)	2.04 (1.63, 2.55)	<0.001
Adjusted OR^3^ (95% CI)	1	1.14 (0.91, 1.43)	1.26 (1.00, 1.59)	2.36 (1.87, 2.97)	<0.001
T2DM vs. (IGR&NGT)					
Cases/control subjects, *n*	238/471	243/493	281/486	482/631	
Crude OR (95% CI)	1	0.98 (0.78, 1.21)	1.14 (0.92, 1.42)	1.51 (1.24, 1.84)	<0.001
Adjusted OR^1^ (95% CI)	1	0.98 (0.78, 1.23)	1.18 (0.94, 1.48)	1.54 (1.25, 1.91)	<0.001
Adjusted OR^2^ (95% CI)	1	0.97 (0.77, 1.22)	1.13 (0.90, 1.42)	1.48 (1.19, 1.83)	<0.001
Adjusted OR^3^ (95% CI)	1	1.04 (0.83, 1.31)	1.27 (1.01, 1.61)	1.72 (1.37, 2.14)	<0.001

Model 1, adjusted for sex, age, body mass index, waist circumference, and family history of diabetes. Model 2, adjusted for smoking status, drinking status, history of hypertension, history of hyperlipidemia, and physical activity based on Model 1. Model 3, adjusted for total protein and albumin levels based on Model 2. Abbreviations: CI, confidence interval; IGR, impaired glucose regulation; NGT, normal glucose tolerance; OR, odds ratio; T2DM, type 2 diabetes mellitus.

**Table 3:** Multiple adjusted odds ratios for plasma transthyretin levels associated with T2DM in subgroups.

Groups	Quartile of Plasma Transthyretin Concentrations (mg/L)				*p* for Trend	*p* for Interaction
	Q1 (Referent)	Q2	Q3	Q4		
	<189.71	189.71–224.44	224.44–264.11	≥264.11		
Sex						0.022
Female (1032)	1	1.20 (0.84, 1.73)	2.13 (1.43, 3.16)	3.43 (2.25, 5.22)	<0.001	
Male (1456)	1	0.98 (0.65, 1.46)	0.98 (0.66, 1.44)	1.88 (1.29, 2.74)	0.001	
Age						0.653
< 60 y (1835)	1	1.03 (0.75, 1.42)	1.28 (0.92, 1.77)	2.22 (1.61, 3.06)	<0.001	
≥ 60 y (653)	1	1.29 (0.78, 2.12)	1.69 (1.03, 2.80)	3.54 (2.05, 6.13)	<0.001	
BMI						0.023
< 24 (1163)	1	1.00 (0.68, 1.45)	1.87 (1.28, 2.73)	2.57 (1.76, 3.76)	<0.001	
≥ 24 (1325)	1	1.30 (0.88, 1.90)	1.13 (0.77, 1.67)	2.63 (1.79, 3.97)	<0.001	
Family historyof diabetes						0.459
No (2045)	1	1.12 (0.84, 1.49)	1.38 (1.03, 1.85)	2.64 (1.97, 3.54)	<0.001	
Yes (443)	1	1.29 (0.65, 2.54)	1.83 (0.88, 3.78)	2.12 (1.07, 4.18)	0.026	
Smoking status						0.007
No (1760)	1	1.25 (0.92, 1.68)	1.67 (1.22, 2.28)	3.35 (2.44, 4.61)	<0.001	
Yes (728)	1	0.79 (0.44, 1.41)	0.83 (0.47, 1.42)	1.26 (0.74, 2.15)	0.091	
Drinking status						0.024
No (1817)	1	1.26 (0.94, 1.68)	1.73 (1.27, 2.35)	3.50 (2.55, 4.81)	<0.001	
Yes (671)	1	0.68 (0.35, 1.32)	0.64 (0.35, 1.18)	1.00 (0.56, 1.76)	0.295	
History of hypertension						0.270
No (1816)	1	1.03 (0.76, 1.39)	1.35 (0.98, 1.84)	2.32 (1.71, 3.16)	<0.001	
Yes (672)	1	1.74 (0.98, 3.10)	1.94 (1.12, 3.36)	3.64 (2.07, 6.40)	<0.001	
History of hyperlipidemia						0.766
No (1733)	1	1.08 (0.80, 1.46)	1.38 (1.01, 1.88)	2.57 (1.88, 3.50)	<0.001	
Yes (755)	1	1.45 (0.82, 2.57)	1.93 (1.09, 3.44)	2.78 (1.59, 4.84)	<0.001	
Physical activity						0.735
No (1529)	1	1.08 (0.76, 1.52)	1.34 (0.95, 1.88)	2.46 (1.74, 3.49)	<0.001	
Yes (959)	1	1.27 (0.83, 1.93)	1.63 (1.05, 2.53)	2.81 (1.83, 4.32)	<0.001	

Adjusted for sex, age, BMI, waist circumference, family history of diabetes, smoking status, drinking status, history of hypertension, history of hyperlipidemia, physical activity, total protein, and albumin levels. Abbreviations: BMI, body mass index; T2DM, type 2 diabetes mellitus.

## Data Availability

The data presented in this study are available on reasonable request from the corresponding author.
